# A Comparison of neoadjuvant chemotherapy and concurrent chemoradiotherapy for for FIGO 2018 stage IB3/IIA2 Cervical squamous cell carcinoma: Long-term efficacy and safety in a resource-limited setting

**DOI:** 10.1371/journal.pone.0319405

**Published:** 2025-03-25

**Authors:** Renxian Xie, Keyan Xie, Xiaoluan Lin, Yanchen Ji, Jianzhou Chen, Chuangzhen Chen

**Affiliations:** 1 Department of Radiation Oncology, Cancer Hospital of Shantou University Medical College, Shantou, P.R. China; 2 Shantou University Medical College, Shantou, P.R. China; Mie University, Graduate School of Medicine, JAPAN

## Abstract

**Purpose:**

The purpose of this research was to evaluate the effectiveness and safety of neoadjuvant chemotherapy plus radical surgery (NCRS) and concurrent chemoradiotherapy (CCRT) based on three-dimensional conformal radiation therapy (3DCRT) for FIGO 2018 stage IB3/IIA2 patients with cervical squamous cell carcinoma in a resource-limited setting.

**Methods:**

The clinical outcomes and incidence of complications in 137 patients who underwent NCRS with those of 163 patients who CCRT based on 3DCRT were compared. Propensity score matching (PSM) analysis was used to match the two groups to enable further statistical comparisons. Survival analysis was performed utilizing Cox proportional hazards regression analyses, Kaplan-Meier curves, and log-rank tests. Furthermore, the incidence of complications between the two groups was also compared using chi-squared tests.

**Results:**

PSM analysis identified 103 matched pairs of patients. The NCRS and CCRT groups exhibited 5-year overall survival (OS) rates of 85.4% and 91.2%, respectively (p=0.19). Additionally, the NCRS and CCRT groups exhibited 5-year disease-free survival (DFS) rates of 76.7% and 89.3% (p=0.02), and the recurrence rates were 20.4% and 9.7% (p=0.03), respectively. However, the CCRT group exhibited a higher incidence of early any-grade complications (79.6% vs 35.9%, p<0.001) and early grade 3 complications (15.5% vs 2.9%, p=0.002) compared to the NCRS group. In terms of overall late complications, there was no significant difference in the incidence between the two groups. Multivariate analysis revealed that stage IIA2 emerged as an independent risk factor for OS (aHR 8.89; p=0.033). Moreover, histologic grade 2–3 (aHR 5.3; p=0.022), stage IIA2 (aHR 2.95; p=0.043), NCRS treatment (aHR 2.41; p=0.012) were identified as independent risk factors for DFS.

**Conclusion:**

In resource-limited settings, for patients with FIGO 2018 stage IB3/IIA2 cervical squamous cell carcinoma, 3DCRT-based CCRT offers superior disease-free survival and reduced recurrence rates compared to NCRS, despite increased early complication rates.

## Introduction

Cervical cancer is a global health concern with a disproportionate impact on middle-aged women, particularly in regions with limited healthcare resources [1–4]. Many developing countries with limited access to healthcare have higher incidence and mortality rates from cervical cancer, and 70% of patients with cervical cancer have local infiltration or metastasis [1–3]. Although advances in screening and human papillomavirus vaccination have contributed to a decline in its incidence in developed countries [5], cervical cancer continues to pose a pressing challenge in less affluent parts of the world. According to the 2018 Federation International of Gynecology and Obstetrics (FIGO) staging, Stage IB3/IIA2 cervical cancer is usually defined as locally advanced cervical cancer (LACC), which requires more aggressive and nuanced treatment approaches than early-stage cancer.

The National Comprehensive Cancer Network (NCCN) recommends concurrent chemoradiotherapy as the standard therapy for LACC. However, the standard treatment for LACC is still controversial, and the patients’ survival rate continues to be discouraging. In regard to this, the concept of neoadjuvant chemotherapy for LACC has been proposed in order to increase the resection rate of patients with LACC and reduce the incidence of postoperative recurrence and metastasis [6]. Neoadjuvant chemotherapy has been documented to improve the 5-year survival rate by approximately 10%-15% via tumor loading reduction, cancer staging degradation and systemic micrometastases suppression [7–9]. Longer disease-free survival (DFS) and overall survival (OS) are observed in NCRS groups than CCRT groups on patients with massive cervical cancer [10–12]. Nevertheless, A randomized controlled trail conducted by Gupta et al. find that cisplatin-based CCRT resulted in superior DFS compared with NCRS in LACC [13]. Some studies indicate that NCRS does not significantly reduce therapeutic complications or prolong 5-year OS compared to CCRT [14]. Additionally, NCRS might lead to poor local tumor control, since neither distant metastasis-free survival nor loco-regional failure-free survival display more satisfactory results compared with CCRT [15]. Therefore, it is still valuable to conduct studies to further clarify the long-term efficacy and safety of NCRS and CCRT for patients with LACC.

The advancement of radiotherapy technology has laid a solid foundation for improving the therapeutic outcomes of cervical cancer. Over the past few decades, radiotherapy has undergone revolutionary changes. From the initial three-dimensional conformal radiotherapy (3DCRT) to the subsequent intensity-modulated radiotherapy (IMRT), image-guided radiotherapy, and biologically guided radiotherapy, these advanced technologies have significantly increased the precision of external beam radiotherapy. They have concentrated the dose to the target area while minimizing the irradiation of vital organs, thereby markedly reducing the incidence of treatment-related adverse reactions. Especially in the treatment of LACC, compared to traditional 3DCRT, IMRT has demonstrated its significant advantage in sparing normal tissues of the patient. It can effectively reduce acute adverse reactions, particularly those affecting the gastrointestinal, genitourinary, and hematopoietic systems, without compromising the local tumor control effect [16,17]. However, despite the advancements in IMRT in many parts of the world, 3DCRT continues to be widely used in resource-limited settings. In settings where only 3DCRT is available, the comparative long-term outcomes, effectiveness and tolerability of these treatments remain uncertain.

This study aims to assess the long-term efficacy and safety outcomes of NCRS versus CCRT based on 3DCRT for patients with FIGO 2018 stage IB3/IIA2 cervical squamous cell carcinoma (CSCC), in the specific context of a resource-constrained environment where advanced RT techniques are unavailable. The findings of this study are anticipated to have crucial implications for clinical decision-making and health policy formulations, potentially enhancing the survival and quality of life for women afflicted with this challenging disease in regions where healthcare resources are scarce.

## Materials and methods

### Data source

The original data are openly accessible on the internet and the authors have given their permission for other researchers to use them, which were accessed on February 1, 2024 [14]. A retrospective cohort study was conducted using the baseline characteristics of the data set. The database file contained the following baseline variables: clinical stage, anemia, tumor diameter, degree of pathological differentiation, and age. The study was conducted in accordance with the Declaration of Helsinki (as revised in 2013).

### Patients

According to the 2018 FIGO classification, the clinical outcomes of patients with CSCC stages IB3 and IIA2 who had treatment at Tianjin Central Hospital of Gynecology Obstetrics (Tianjin, China) between January 2011 and December 2016 were retrospectively analyzed in this study. Histological confirmation was obtained through cervical biopsies. In this research, cases with a pathologic diagnosis of adenocarcinoma or adenosquamous carcinoma and an interval of less than 6 months between the end of treatment and the beginning of the study were excluded from the study. The primary method for assessing lymph node metastasis prior to treatment was through preoperative imaging, specifically abdominopelvic magnetic resonance imaging or computed tomography. These imaging modalities were utilized to screen for any suspicious lymph node enlargement or abnormal morphological features that might indicate potential metastasis. The cases of positive lymph nodes in frozen sections analysis or postoperative pathological analysis were also excluded from our study.

### Treatment procedures

Paclitaxel (135–155 mg/m^2^, day 1) and cisplatin (60 mg/m^2^, day 1)/paclitaxel (135–155 mg/m^2^, day 1) and carboplatin(area under the concentration-time curve 5.0–7.5, day 1) were given in two cycles at 21-day intervals to the NCRS group [18]. After receiving neoadjuvant chemotherapy, the patients underwent radical hysterectomy and lymphadenectomy via laparotomy or laparoscopy, on average, seven days later. Lymphadenectomy was used in this study, i.e., at least 20 or more lymph nodes were cleared to properly assess true pelvic lymph node metastasis. Analysis of frozen sections was done for the lymphadenectomy’s external iliac lymph nodes. Patients in the CCRT group received concurrent chemotherapy and external pelvic radiotherapy together with brachytherapy. The entire pelvis received 45 Gy of external beam radiation (EBRT) in 25 fractions utilizing the four-field box technique based on 3DCRT and 60Co external beam. Using 192Ir sources, high-dose-rate brachytherapy was carried out weekly for four weeks in a row, with a total dose of 35-45 Gy (2-Gy daily fractions (EQD2), assuming an α/β ratio of 10 Gy) at point A, which results in a total prescribed dose of 80-90 Gy EQD2 at point A. Concurrent chemotherapy was initiated at the beginning of EBRT. At intervals of 21 days, patients were administered cisplatin (50 mg/m2, day1) and paclitaxel (135–175 mg/m2, day1) in three or four cycles [19]. None of these patients had received adjuvant chemotherapy or adjuvant chemoradiotherapy after surgery or CCRT.

### Follow-up

Specific examinations for periodic follow-up visits, including abdominopelvic magnetic resonance imaging (MRI) or computed tomography (CT). For patients in the NCRS group, tumor size was initially estimated using imaging studies such as MRI or CT scans. Postoperatively, the final tumor size was confirmed through pathological examination of the surgical specimens. The pathologists measured the greatest dimension of the tumor in the resected tissue to provide an accurate assessment of the tumor size. For patients in the CCRT group, tumor size was primarily assessed using imaging studies (MRI or CT) as these patients did not undergo surgery. The imaging studies were used to monitor the response to treatment and to assess the tumor size at various time points during the treatment course.

Following treatment, patients in the CCRT group underwent a pelvic exam as part of their follow-up, with the purpose of evaluating their disease status based on the NCCN criteria [20,21]. Safety was evaluated by considering complications based on the Chassagne glossary [22]. Complications that arise during the treatment period or within three months post-treatment completion are categorized as early complications and are systematically evaluated using the Common Terminology Criteria of Adverse Event (CTCAE v5.0). Late complications are identified as any adverse events occurring beyond 91 days after the conclusion of treatment, and are assessed using the Radiation Therapy Oncology Group/European Organization of Research and Treatment of Cancer (RTOG/EORTC) late toxicity criteria.

### Propensity score matching

The propensity score matching (PSM) method is extensively employed in observational studies to reduce selection bias [23,24]. In our research, we utilized PSM to equilibrate baseline covariates between the NCRS and CCRT group. This was achieved through a 1:1 nearest neighbor matching approach, ensuring the balance of clinical baseline characteristics such as age, tumor diameter, anemia status, tumor clinical stage, and pathological grade. A caliper value of 0.02 was established for the matching process.

### Statistical analysis

A Mann-Whitney U test and a two-tailed Student’s t test was used to assess differences in clinical characteristics between the NCRS and CCRT group. The time interval between the start of treatment and the date of the first recorded indication of relapse at any site (local recurrence, metastasis, or both) or death was defined as DFS, and OS duration was computed from the start of treatment until the patient’s passing. The OS and DFS analysis was conducted using Kaplan-Meier curves, and the statistical significance was evaluated using the log-rank test. For both univariate and multivariate analyses of OS and DFS for all patients, Cox proportional hazards regression analyses with 95% confidence intervals (CIs), Log-rank test, and adjust hazard ratio (aHR) were utilized. Additionally, chi-squared tests were employed to compare the incidence of complications between the two groups. Statistical significance was defined as a p-value <0.05. We used IBM SPSS Statistics (version R26.0.0.2) and Free Statistics software versions 1.9 for statistical analysis.

## Results

### Patient characteristics

**Fig 1:**
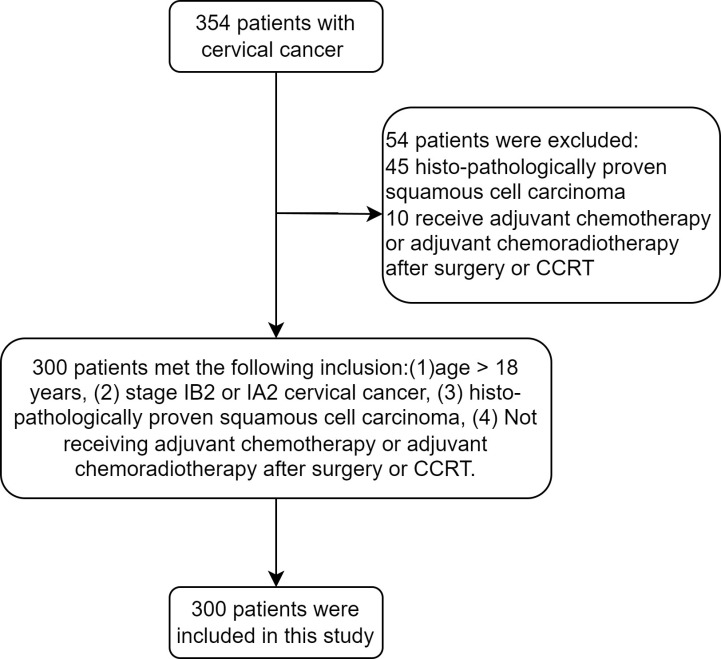
Flow-chart of the patient selection procedure.

A flowchart for the patient selection process is displayed in Fig 1. Following careful consideration of the inclusion and exclusion criteria, 137 of the 300 patients who were initially included were in the NCRS group, and 163 were in the CCRT group. None of these patients had received adjuvant chemotherapy, adjuvant chemoradiotherapy, or salvage hysterectomy after radical surgery or CCRT. The clinicopathological features of the NCRS and CCRT groups before and after PSM are listed in Table 1. Compared to the CCRT group, the NCRS group had a lower percentage of patients with stage IIA2 before to PSM. Once age, anemia, tumor diameter, degree of pathological differentiation, and clinical stage were matched 1:1, 103 patients from each group with balanced baseline characteristics made up each group. According to the Querleu-Morrow classification of radical hysterectomy [25], 20 patients in the NCRS group underwent laparoscopy interventions, all of which were classified as type C2. Additionally, 83 patients underwent laparotomy interventions, with three patients classified as type B2 and 80 patients classified as type C2.

**Table 1:** Patient demographics and baseline characteristics.

Characteristics	Before matching			After matching		
	CCRT group, N = 163	NCRS group, N = 137	p-value	CCRT group, N = 103	NCRS group, N = 103	p-value
Age (Mean ± SD)	47.7 ± 8.7	46.5 ± 8.7	0.268	46.4 ± 8.1	45.5 ± 8.3	0.445
Initial tumor size (Mean ± SD) (cm)	4.31 ± 0.67	4.19 ± 0.51	0.073	4.11 ± 0.33	4.17 ± 0.53	0.307
Anemia before treatment, n (%)			0.311			0.223
No	136 (83.4%)	120 (87.6%)		92 (89.3%)	86 (83.5%)	
Yes	27 (16.6%)	17 (12.4%)		11 (10.7%)	17 (16.5%)	
FIGO 2018 stage, n (%)			<0.001			0.305
Ib3	35 (21.5%)	73 (53.5%)		32 (31.1%)	39 (37.9%)	
IIa2	128 (78.5%)	64 (46.7%)		71 (68.9%)	64 (62.1%)	
Histologic grade, n (%)			0.341			0.738
G1	51 (31.3%)	36 (26.3%)		24 (23.3%)	22 (21.4%)	
G2-3	112 (68.7%)	101 (73.7%)		79 (76.7%)	81 (78.6%)	

NCRS: Neoadjuvant chemotherapy followed by radical surgery; CCRT, concurrent chemoradiotherapy; FIGO: International Federation of Gynecology and Obstetrics; SD: Standard Deviation.

### Long-term efficacy

**Fig 2:**
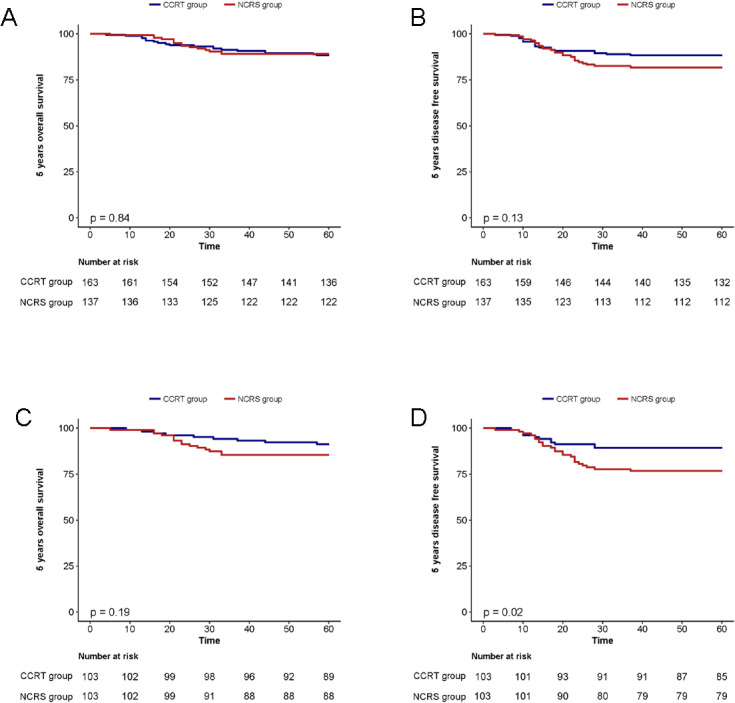
A, B Kaplan–Meier analysis estimates of the 5 year OS and DFS rate in the NCRS and CCRT groups before PSM. C, D Kaplan–Meier analysis estimates of the 5 year OS and DFS rate in the NCRS and CCRT groups after PSM.

The duration of median follow-up was 76 months with no patients lost to follow-up. Among the NCRS group, which consisted of 137 patients, recurrence was observed in 22 patients (16.1%), while 16 patients (11.7%) died. On the other hand, in the CCRT group, encompassing 163 patients, recurrence and mortality rates were identical, with 19 patients (11.7%) experiencing recurrence and 19 patients (11.7%) passing away. Prior to PSM, the recurrence rates between the two groups did not exhibit significant differences. The patterns of recurrence for both groups, analyzed both before and after PSM, are depicted in Table 2. Kaplan-Meier survival analysis demonstrated that the 5-year OS rates were 89.1% in the NCRS group and 91.2% in the CCRT group, with no significant difference observed (p = 0.84; Fig 2A). Similarly, the 5-year DFS rates were 81.8% for the NCRS group and 81.0% for the CCRT group, indicating no significant difference (p = 0.13; Fig 2B). Multivariate analysis revealed that initial tumor size >4.3 cm(aHR 3.72; p = 0.047), stage IIA2(aHR 7.05; p = 0.002) emerged as an independent risk factor for OS, while Age >46 years(aHR 2.06; p = 0.021), stage IIA2 (aHR 5.55; p = 0.004) and NCRS treatment (aHR 1.55; p = 0.01) were identified as independent risk factors for DFS. Multivariate analyses of the 5 year OS rate and DFS rate by Cox proportional hazards regression models before and after PSM are depicted in Table 3.

**Table 2:** Pattern of recurrence in the NCRS and CCRT groups before and after PSM.

Recurrence Site	Before matching			After matching		
	NCRS group(n=137)	CCRT group(n=163)	p-value	NCRS group(n=103)	CCRT group(n=103)	p-value
**Recurrence, n (%)**	22 (16.1)	19 (11.7)	0.269	21 (20.4)	10 (9.7)	0.032
**Local**	8 (5.8)	3 (1.8)	0.066	8 (7.8)	3 (2.9)	0.121
lower vaginal	3 (2.2)	1 (0.6)	0.335	3 (2.9)	1 (1)	0.621
parametrial	4 (2.9)	2 (1.2)	0.417	4 (3.9)	2 (1.9)	0.683
bladder	1 (0.7)	0 (0)	0.457	1 (1)	0 (0)	1
**Distant, n (%)**	13 (9.5)	15 (9.2)	0.932	12 (11.7)	6 (5.8)	0.139
Supraclavicular lymph node metastasis	4 (2.9)	2 (1.2)	0.417	3 (2.9)	2 (1.9)	1
Isolated pulmonary metastatic (≤3 lesions)	0 (0)	5 (3.1)	0.065	0 (0)	0 (0)	1
Multiple pulmonary metastatic (>3 lesions)	9 (6.6)	8 (4.9)	0.535	9 (8.7)	4 (3.9)	0.152
**Local plus distant, n (%)**	1 (0.7)	1 (0.6)	1	1 (1)	1 (1)	1

NCRS: Neoadjuvant chemotherapy followed by radical surgery; CCRT, concurrent chemoradiotherapy; FIGO: International Federation of Gynecology and Obstetrics

**Table 3:** Multivariate analyses of the OS rate and DFS rate by Cox proportional hazards regression models in the NCRS and CCRT groups before and after PSM.

Characteristic	Before matching				After matching			
	OS		DFS		OS		DFS	
	aHR(95% CI)	p-value	aHR(95%CI)	p-value	aHR(95%CI)	p-value	aHR(95%CI)	p-value
Age >46 years	1.21 (0.61~2.39)	0.593	2.06 (1.12~3.8)	0.021	1.79 (0.79~4.06)	0.161	0.85 (0.44~1.63)	0.623
Anemia before treatment	0.83 (0.3~2.29)	0.724	0.6 (0.33~1.08)	0.09	0.69 (0.2~2.34)	0.549	1.6 (0.72~3.56)	0.25
Initial tumor size >4.3 cm	3.72 (1.02~13.58)	0.047	1.4 (0.67~2.9)	0.37	8.89 (0.91~87.14)	0.061	3.39 (0.94~12.26)	0.063
Histologic grade G2-3	1.71 (0.74~3.96)	0.207	2.45 (0.96~6.29)	0.062	1.74 (0.59~5.17)	0.318	5.3 (1.27~22.09)	0.022
FIGO 2018 stage (IB3 vs. IIA2)	7.05 (2.07~23.99)	0.002	5.55 (1.71~17.94)	0.004	8.89 (1.19~66.35)	0.033	2.95 (1.03~8.46)	0.043
Treatment (NCRS vs. CCRT)	1.55 (0.77~3.12)	0.224	3 (1.31~6.9)	0.01	2.11 (0.93~4.83)	0.076	2.41 (1.21~4.79)	0.012

NCRS: Neoadjuvant chemotherapy followed by radical surgery; CCRT, concurrent chemoradiotherapy; FIGO: International Federation of Gynecology and Obstetrics;OS: overall survival; DFS: disease-free survival; aHR: adjust hazard radio; CI: confidence interval

Following 1:1 case matching to equilibrate baseline characteristics, the observed recurrence rates were 20.4% in the NCRS group and 9.7% in the CCRT group, revealing a statistically significant difference (p = 0.032). Analysis of recurrence patterns demonstrated that the CCRT group exhibited reduced incidences of recurrences in comparison to those observed in the NCRS group. The 5-year OS rates for the NCRS and CCRT groups were 85.4% and 91.2%, respectively (p = 0.19; Fig 2C). However, the 5-year DFS rates were 76.7% for the NCRS group and 89.3% for the CCRT group, showing a statistically significant difference (p = 0.02; Fig 2D). Multivariate analysis revealed that stage IIA2 emerged as an independent risk factor for OS (aHR 8.89; p = 0.033). Moreover, histologic grade 2–3 (aHR 5.3; p = 0.022), 2018 FIGO stage IIA2 (aHR 2.95; p = 0.043), NCRS treatment (aHR 2.41; p = 0.012) were identified as independent risk factors for DFS.

Therefore, based on multivariate COX regression analyses, the CCRT group had significantly higher DFS rates compared to the NCRS group. Additionally, the CCRT group showed lower rates of recurrence, indicating better long-term efficacy in a resource-limited setting where only a 3DCRT was available.

### Safety

Early and late complications before and after PSM are listed in Table 4. For grade early 1–2 complications, 17 patients undergoing NCRS treatment experienced various degrees of myelosuppression, which included anemia, thrombocytopenia, and neutropenia, in contrast to 55 patients who received CCRT treatment (12.4% vs. 33.7%, respectively; p < 0.001). Moreover, 11 patients treated with NCRS exhibited a range of gastrointestinal reactions, encompassing vomiting, diarrhea, transient gastrointestinal bleeding, and intestinal obstruction, compared to 72 patients in the CCRT group (8.0% vs. 44.2%, respectively; p < 0.001), highlighting significant differences in the incidence of these early complications between the two groups. Furthermore, urinary complications, including incontinence, urgency, and dysuria of varying degrees, were recorded in 16 patients versus 59 patients treated with CCRT, representing statistically significant differences in occurrence rates between groups (11.7% vs. 36.2%; p < 0.001). In addition, grade 3 early complications were observed in three patients in the NCRS group as opposed to 29 patients in the CCRT group (2.2% vs. 17.8%, respectively; p < 0.001).

**Table 4:** Early and late complications in the NCRS and CCRT groups before and after PSM.

Result (n)	Before matching			After matching		
	NCRS group(n=137)	CCRT group(n=163)	p-value	NCRS group(n=103)	CCRT group(n=103)	p-value
**Early complications, n(%)**	38 (27.7)	127 (77.9)	<0.001	37 (35.9)	82 (79.6)	<0.001
**Grade 1-2**	36 (26.3)	103 (63.2)	<0.001	35 (34)	70 (68)	<0.001
myelosuppression	17 (12.4)	55 (33.7)	<0.001	17 (16.5)	30 (29.1)	0.031
Gastrointestinal	11 (8)	72 (44.2)	<0.001	11 (10.7)	50 (48.5)	<0.001
Urinary	16 (11.7)	59 (36.2)	<0.001	15 (14.6)	49 (47.6)	<0.001
**Grade 3**	3 (2.2)	29 (17.8)	<0.001	3 (2.9)	16 (15.5)	0.002
myelosuppression	0 (0)	9 (5.5)	0.004	0 (0)	8 (7.8)	0.007
Gastrointestinal	3 (2.2)	14 (8.6)	0.017	3 (2.9)	5 (4.9)	0.721
Urinary	0 (0)	6 (3.7)	0.033	0 (0)	3 (2.9)	0.246
**Late complications, n(%)**	48 (35.0)	57 (35.0)	0.99	37 (35.9)	30 (29.1)	0.298
**Grade 1-2**	46 (33.6)	55 (33.7)	0.976	35 (34)	28 (27.2)	0.29
Gastrointestinal	0 (0)	18 (11)	<0.001	0 (0)	7 (6.8)	0.014
Urinary	34 (24.8)	3 (1.8)	<0.001	31 (30.1)	2 (1.9)	<0.001
Symptomatic vaginal stenosis	0 (0)	27 (16.6)	<0.001	0 (0)	12 (11.7)	<0.001
pelvic lymphedema	12 (8.8)	25 (15.3)	0.084	4 (3.9)	14 (13.6)	0.014
**Grade 3**	2 (1.5)	3 (1.8)	1	2 (1.9)	2 (1.9)	1
Gastrointestinal	0 (0)	3 (1.8)	0.253	0 (0)	2 (1.9)	0.498
pelvic lymphedema	2 (1.5)	0 (0)	0.208	2 (1.9)	0 (0)	0.498

NCRS: Neoadjuvant chemotherapy followed by radical surgery; CCRT, concurrent chemoradiotherapy; FIGO: International Federation of Gynecology and Obstetrics.

Regarding grade 1-2 late complications, in the CCRT group, pelvic lymphedema was reported in 25 patients (15.3%), symptomatic vaginal stenosis in 27 patients (16.6%), persistent gastrointestinal bleeding in 18 patients (11%), and hematuria in 3 patients (1.8%). For the grade 3 late complications, three cases (1.8%) necessitated partial intestinal resection due to enteric necrosis. In contrast, NCRS treatment resulted in 48 cases (35.0%) of grade 1-2 late complications, with urinary issues (urgency, dysuria, and ureterohydronephrosis) being the most prevalent in 34 patients (24.8%), followed by pelvic lymphedema in 12 cases (8.8%). Additionally, two cases (1.5%) of grade 3 pelvic lymphedema were reported. Despite these findings, no significant differences were observed between the two groups concerning the incidence of late complications. Moreover, no grade 4 complication was observed in either treatment group.

Results were similar before and after PSM, with a higher incidence of early complications (including myelosuppression, gastrointestinal and urinary complications) in the CCRT group compared with the NCRS group. This difference was observed in both any-grade complications (79.6% vs 35.9%, p < 0.001) and grade=3 complications (15.5% vs 2.9%, p = 0.002). However, there was no statistically significant differences observed between the two groups in relation to late complications.

### NCRS group (patients with laparotomy) vs. CCRT group

**Fig 3:**
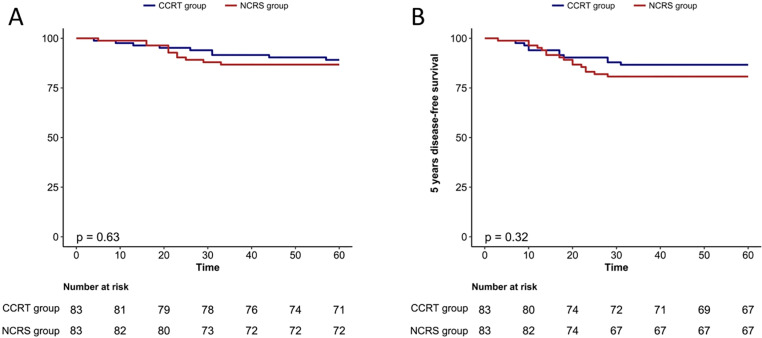
A, B Kaplan–Meier analysis estimates of the 5 year OS and DFS rate in the NCRS (patients with laparotomy) and CCRT groups after PSM.

Given the evidence from large-scale randomized clinical trials, such as the LACC trial, indicating that laparoscopy is associated with a poorer prognosis compared to laparotomy [26], we conducted a comprehensive analysis to compare the efficacy outcomes between patients in the NCRS group who underwent laparotomy and those in the CCRT group. PSM analysis identified 83 matched pairs of patients. The NCRS (patients with laparotomy) and CCRT groups exhibited 5-year OS rates of 94.4% and 96.1%, respectively (p=0.63; Fig 3A). Additionally, the NCRS and CCRT groups exhibited 5-year DFS rates of 80.7% and 86.7% (p=0.32; Fig 3B), and the recurrence rates were 15.7% and 10.8% (p=0.36), respectively. The patterns of recurrence in the NCRS and CCRT groups after PSM are depicted in Table 5. Multivariate analysis revealed that stage IIA2 emerged as an independent risk factor for OS (aHR 15.8; p=0.017). Moreover, histologic grade 2–3 (aHR 4.05; p=0.023) was identified as an independent risk factor for DFS. Multivariate analyses of the 5-year OS rate and DFS rate by Cox proportional hazards regression models before and after PSM are depicted in Table 6.

**Table 5:** Pattern of recurrence in the NCRS group (patients with laparotomy) and CCRT group after PSM.

Recurrence Site	After matching		
	NCRS group(n=83)	CCRT group(n=83)	p-value
**Recurrence, n (%)**	13 (15.7)	9 (10.8)	0.36
**Local**	4 (4.8)	2 (2.4)	0.682
lower vaginal	1 (1.2)	1 (1.2)	1
parametrial	2 (2.4)	1 (1.2)	1
bladder	1 (1.2)	0 (0)	1
**Distant, n (%)**	8 (9.6)	6 (7.2)	0.576
Supraclavicular lymph node metastasis	1 (1.2)	2 (2.4)	1
Isolated pulmonary metastatic (≤3 lesions)	0 (0)	1 (1.2)	1
Multiple pulmonary metastatic (>3 lesions)	7 (8.4)	3 (3.6)	0.192
**Local plus distant, n (%)**	1 (1.2)	1 (1.2)	1

NCRS: Neoadjuvant chemotherapy followed by radical surgery; CCRT, concurrent chemoradiotherapy; FIGO: International Federation of Gynecology and Obstetrics.

**Table 6:** Multivariate analyses of the OS rate and DFS rate by Cox proportional hazards regression models in the NCRS group (patients with laparotomy) and CCRT group after PSM.

Characteristic	After matching			
	OS		DFS	
	aHR(95%CI)	p-value	aHR(95%CI)	p-value
Age >46 years	1.84 (0.75~4.54)	0.186	0.86 (0.4~1.83)	0.698
Anemia before treatment	0.51 (0.1~2.54)	0.413	1.67 (0.61~4.55)	0.315
Initial tumor size >4.3 cm	7.29 (0.74~72.13)	0.089	3.08 (0.65~14.51)	0.155
Histologic grade G2-3	1.77 (0.61~5.13)	0.294	4.05 (1.21~13.54)	0.023
FIGO 2018 stage (IB3 vs. IIA2)	15.8 (1.64~152.54)	0.017	3.37 (0.91~12.55)	0.07
Treatment (NCRS vs. CCRT)	1.57 (0.65~3.8)	0.316	1.61 (0.77~3.37)	0.208

NCRS: Neoadjuvant chemotherapy followed by radical surgery; CCRT, concurrent chemoradiotherapy; FIGO: International Federation of Gynecology and Obstetrics;OS: overall survival; DFS: disease-free survival; aHR: adjust hazard radio; CI: confidence interval.

### Sensitivity analysis

In order to evaluate the robustness of our study’s results, sensitivity analysis was performed. Patients who were initially excluded due to adenocarcinoma (S1,S2 Fig, S1 Table), postoperative adjuvant chemotherapy or chemoradiotherapy (S3, S4 Fig, S2 Table), and non-squamous cell carcinoma (S5, S6 Fig, S3 Table) were reintegrated into the analysis. The results of the sensitivity analyses were in concordance with the primary study outcomes, demonstrating that NCRS treatment is an independent prognostic factor for DFS rather than OS. For further details, refer to the **supplementary materials**.

## Discussion

Our findings revealed no significant difference in 5-year OS and DFS rates between the NCRS and CCRT groups in the pre-PSM population. However, post-PSM analysis indicated a statistically significant improvement in DFS for patients treated with CCRT, suggesting a potentially more robust long-term treatment outcome. Additionally, the CCRT group showcased lower incidence rates of recurrence, further underscoring the treatment’s efficacy.

However, this is not consistent with the results of some previous retrospective studies. A retrospective study by Lee et al. comparing the efficacy of 85 patients treated with NCRS versus 358 patients treated with CCRT in patients with stage IB-IIB cervical cancer showed that there was no significant difference between the two treatment regimens in terms of 5-year local tumor recurrence rates [27]. In addition, another retrospective study by Yin et al., encompassing 476 patients with stage IB–IIB cervical cancer, revealed that the 5-year OS and DFS rates among patients who underwent NCRS were significantly superior to those of the patients receiving CCRT [28]. The outcomes of the aforementioned retrospective analyses, focused on long-term effects, consistently indicate that the prognosis for patients treated with NCRS is comparable to, if not superior to, that of patients who underwent CCRT for stage IB-IIB patients. The divergence of our results from those reported in earlier research may stem from our rigorous mitigation of potential bias via PSM analysis, taking into account variables such as age, presence of anemia, tumor size, level of pathological differentiation, and clinical stage.

Anemia has been increasingly found to be a poor prognostic factor in cervical cancer, especially in patients with locally advanced cervical cancer [29–34]. The primary symptom of most cervical cancer patients is irregular vaginal bleeding, which can easily lead to chronic blood loss and result in cancer-related anemia. And anemia affects the efficacy of CCRT in cancer patients. Research has shown that anemic patients have a poorer response to CCRT compared to non-anemic patients [35,36]. The impact of anemia on CCRT for cervical cancer patients is primarily reflected in the following aspects [37]: The anemic state facilitates the creation of a hypoxic environment in tumor tissue, which can reduce the sensitivity of tumor cells to radiotherapy and certain chemotherapeutic drugs (such as platinum-based drugs). This is because in a hypoxic environment, the DNA damage repair ability of tumor cells is enhanced, thereby reducing cell apoptosis induced by radiotherapy and chemotherapy. A decrease in hemoglobin concentration can affect the oxygen content of tumor tissue, leading to an increase in the proportion of hypoxic cells. Oxygen is one of the most common radiosensitizers, and the number of hypoxic cells and cellular oxygen content can influence the sensitivity of tumor cells to radiation, affecting the killing effect of radiation on tumor cells. This can lead to radiation resistance in tumor cells and poorer treatment outcomes for patients [38]. Previous experimental results have also shown that reducing the oxygen partial pressure in tumor tissue significantly decreases its sensitivity to radiotherapy, increasing radiation resistance [39–41]. Furthermore, anemia can lead to poor blood circulation, affecting the distribution and metabolism of chemotherapeutic drugs in the patient’s body. When hemoglobin levels decrease, the ability of patients to transport chemotherapeutic drugs also decreases, resulting in a lower effective drug concentration needed to kill tumor cells, thus promoting tumor progression. Theoretically, tumor cell survival and proliferation require a good oxygen supply and nutrient delivery. Anemia can lead to hypoxia and nutrient deficiency in tumor tissue, further inducing a more aggressive phenotype and abnormal angiogenesis. Normal tissues can correct their mild or moderate anemia symptoms through compensatory mechanisms such as increasing tissue perfusion. However, this compensatory mechanism is not feasible in tumor tissue, possibly due to the atypical vascular network present in tumor tissue [42]. Additionally, anemia can exacerbate some complications during radiotherapy and chemotherapy, such as fatigue, low immune function, and susceptibility to infection. These complications may force clinicians to delay or reduce the dose of radiotherapy and chemotherapy, thereby affecting treatment efficacy. Anemia is often accompanied by symptoms such as fatigue and shortness of breath, which can significantly reduce patients’ quality of life and affect treatment compliance. A previous study retrospectively analyzed the clinical data of 257 LACC patients, exploring the prognostic impact of anemia on these patients after receiving radical radiotherapy and chemotherapy [43]. The results showed that patients with anemia before or during treatment had significantly lower 5-year OS and PFS rates compared to patients without anemia. It’s worth noting that patients who were anemic before treatment but had their hemoglobin levels corrected to normal through treatment showed significant improvement in 5-year OS and PFS compared to patients who remained anemic throughout treatment. Therefore, anemia before and during treatment is considered an independent adverse prognostic factor for LACC patients. Patients with stage IB3 and IIA2 have large tumors, complex blood supply, and a large number of anoxic cells in the tumor, which are insensitive to radiotherapy, while patients with anemia will lead to further anoxia at the site of radiotherapy, thus affecting the therapeutic efficacy of concurrent radiochemotherapy [44]. Based on this, we matched the presence of anemia as a confounder to reduce the effect of confounders. Given the role of anemia in potentially modulating treatment responses, further investigations considering the anemia status of patients could enrich our understanding of these outcomes.

The results of a randomized clinical trial conducted by Gupta et al. that included 633 patients with stage IB2-IIB CSCC are similar to our results [13]. This study revealed that patients treated with CCRT demonstrated a notably higher 5-year DFS rate in comparison to those who received NCRS. However, no significant differences were observed in the 5-year OS rate between the two groups. It’s worth noting that 68 patients (21.5%) were transitioned to definitive CCRT due to either presurgical reassessment or the discovery of intraoperative unresectable disease in the NCRS group. Additionally, 42 patients (13.3%) underwent postoperative adjuvant chemoradiotherapy, and 31 patients (9.8%) received postoperative adjuvant radiotherapy. These treatment modifications might have introduced bias into the study’s outcomes. In contrast, none of the patients included in our study had received adjuvant chemotherapy or adjuvant chemoradiotherapy after surgery or CCRT.

Due to evidence from large-scale randomized clinical trials, such as the LACC trial [26], indicating that laparoscopic surgery is associated with a poorer prognosis compared to open surgery, we conducted a comprehensive analysis to compare the efficacy outcomes between patients in the NCRS group who underwent laparotomy and those in the CCRT group. Our findings demonstrated that the prognosis of the NCRS group with open surgery was comparable to that of the CCRT group in terms of OS, PFS, and recurrence rate. This suggests that for patients with stage IB3/IIA2 CSCC, NCRS with open surgery may serve as an alternative to CCRT when radiotherapy is not feasible. However, these results require further validation through clinical studies with larger sample sizes.

The safety profile analysis of the patients in both groups also merits attention, particularly reflecting on early and late complications associated with treatments. In the NCRS group, the primary treatment-related complications were late urinary side effects, with urethral stricture being the most common. The incidence of urethral stricture in the NACT-RS group underscores the need for careful surgical technique and postoperative management to minimize the risk of such complications. Additionally, patients in the NACT-RS group may require long-term follow-up and potential interventions to manage urethral strictures, which can impact their quality of life. Remarkably, the CCRT group had a higher incidence of grade 1-2 complications, including myelosuppression, gastrointestinal, symptomatic vaginal stenosis, pelvic lymphedema, and urinary complications compared to the NCRS group. This is due to the fact that these areas adjacent to the cervix also receive a certain dose of radiation while undergoing radiation therapy with the 3DCRT technique, which contributes to the occurrence of these treatment-related side effects. Emerging technologies, such as IMRT, hold considerable promise in substantially mitigating the toxicity associated with radiotherapy treatments [45]. However, despite the advancement to IMRT in many parts of the world, 3DCRT continues to be widely used in resource-limited settings.

Despite the impressive results, we acknowledge certain limitations in this study. First, the retrospective nature of the research limits the ability to control confounding variables and potential biases inherent in such studies. Nonetheless, efforts were made to minimize apparent bias through Cox proportional hazards regression models and 1:1 case matching. In addition, this study lacked detailed data on relapse treatment. This limitation prevented us from fully analyzing relapse treatment and its impact on patient prognosis. This was a small-sample retrospective study that included only patients with squamous cervical cancer and may not be able to generalize the findings to patients with cervical adenocarcinoma or adenosquamous carcinoma. Future studies, preferably prospective and randomized, are encouraged to explore the comparative effectiveness and safety of NCRS and CCRT in a broader patient population and with more precise patient stratification.

In summary, our study suggests that CCRT based on 3DCRT offers an improved long-term survival benefit compared to NCRS in patients with stage IB3/IIA2 CSCC. This treatment may represent a valuable option, especially in resource-limited settings where the balance between efficacy, safety, and resource allocation must be carefully weighed.

## Conclusion

In resource-limited settings, for patients with FIGO 2018 stage IB3/IIA2 cervical squamous cell carcinoma, 3DCRT-based CCRT offers superior disease-free survival and reduced recurrence rates compared to NCRS, despite increased early complication rates.

## Supporting information

S1 Fig Patients with squamous or adenocarcinoma_K-M curve of the 5 year DFS after PSM. (TIF)

S2 Fig Patients with squamous or adenocarcinoma_K-M curve of the 5 year OS after PSM. (TIF)

S3 Fig Patients with or without adjuvant therapy_K-M curve of the 5 year DFS after PSM. (TIF)

S4 Fig Patients with or without adjuvant therapy_K-M curve of the 5 year OS after PSM. (TIF)

S5 Fig Patients with all pathology types_K-M curve of the 5 year DFS after PSM. (TIF)

S6 Fig Patients with all pathology types_K-M curve of the 5 year OS after PSM. (TIF)

S1 Table Multivariate analyses of the OS rate and DFS rate by Cox proportional hazards regression models before and after PSM for patients with squamous or adenocarcinoma. (DOCX)

S2 Table Multivariate analyses of the OS rate and DFS rate by Cox proportional hazards regression models before and after PSM for patients with or without adjuvant therapy. (DOCX)

S3 Table Multivariate analyses of the OS rate and DFS rate by Cox proportional hazards regression models before and after PSM for patients with all pathology types. (DOCX)
