# Supplementary figures and images for: A Comparison of neoadjuvant chemotherapy and concurrent chemoradiotherapy for for FIGO 2018 stage IB3/IIA2 Cervical squamous cell carcinoma: Long-term efficacy and safety in a resource-limited setting
Source: PLoS One. 2025 Mar 25;20(3):e0319405. doi: 10.1371/journal.pone.0319405 (PMC11936288; doi:10.1371/journal.pone.0319405)

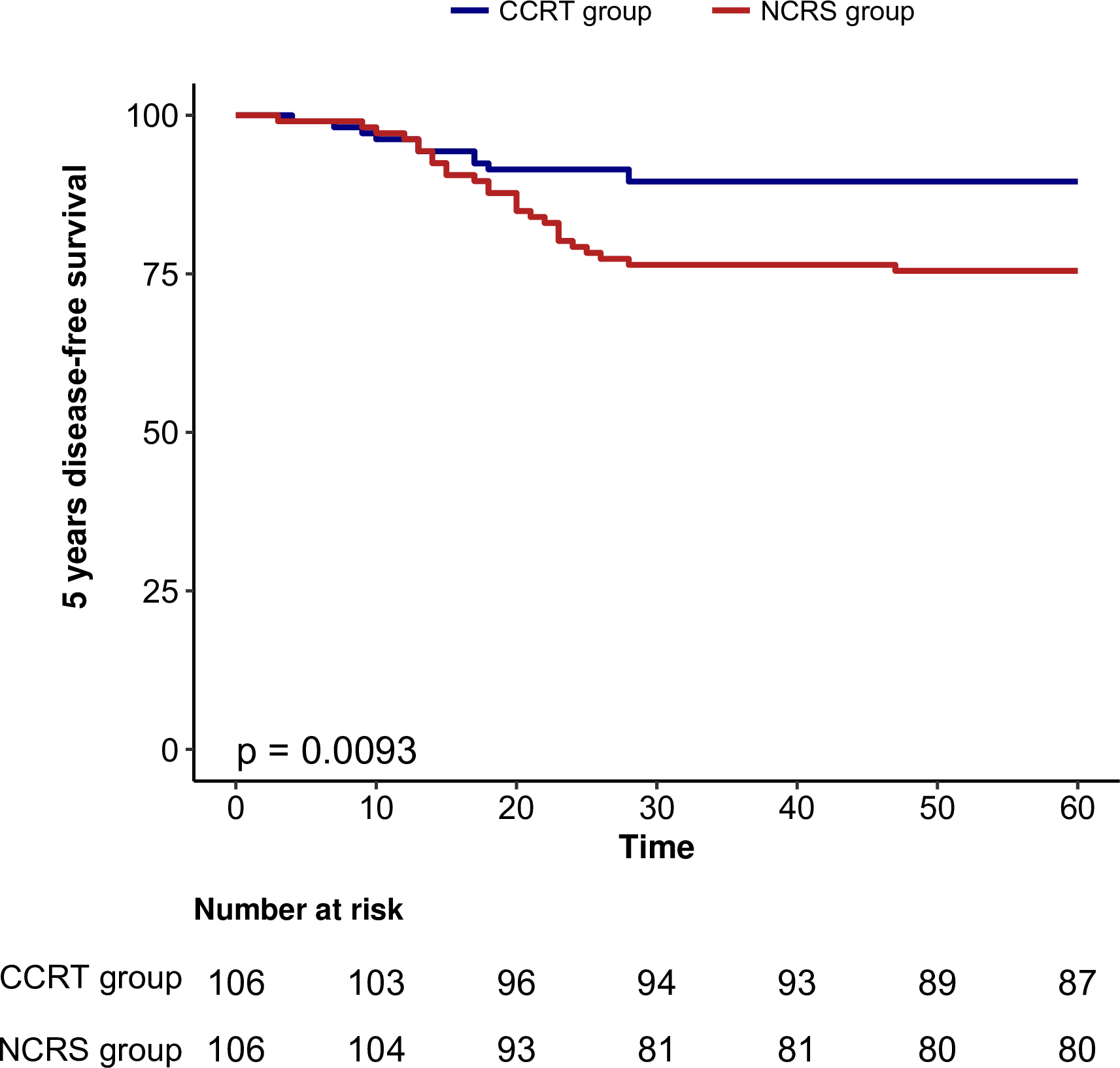

Supplement: S1 Fig — (TIF) [file pone.0319405.s001.tif]

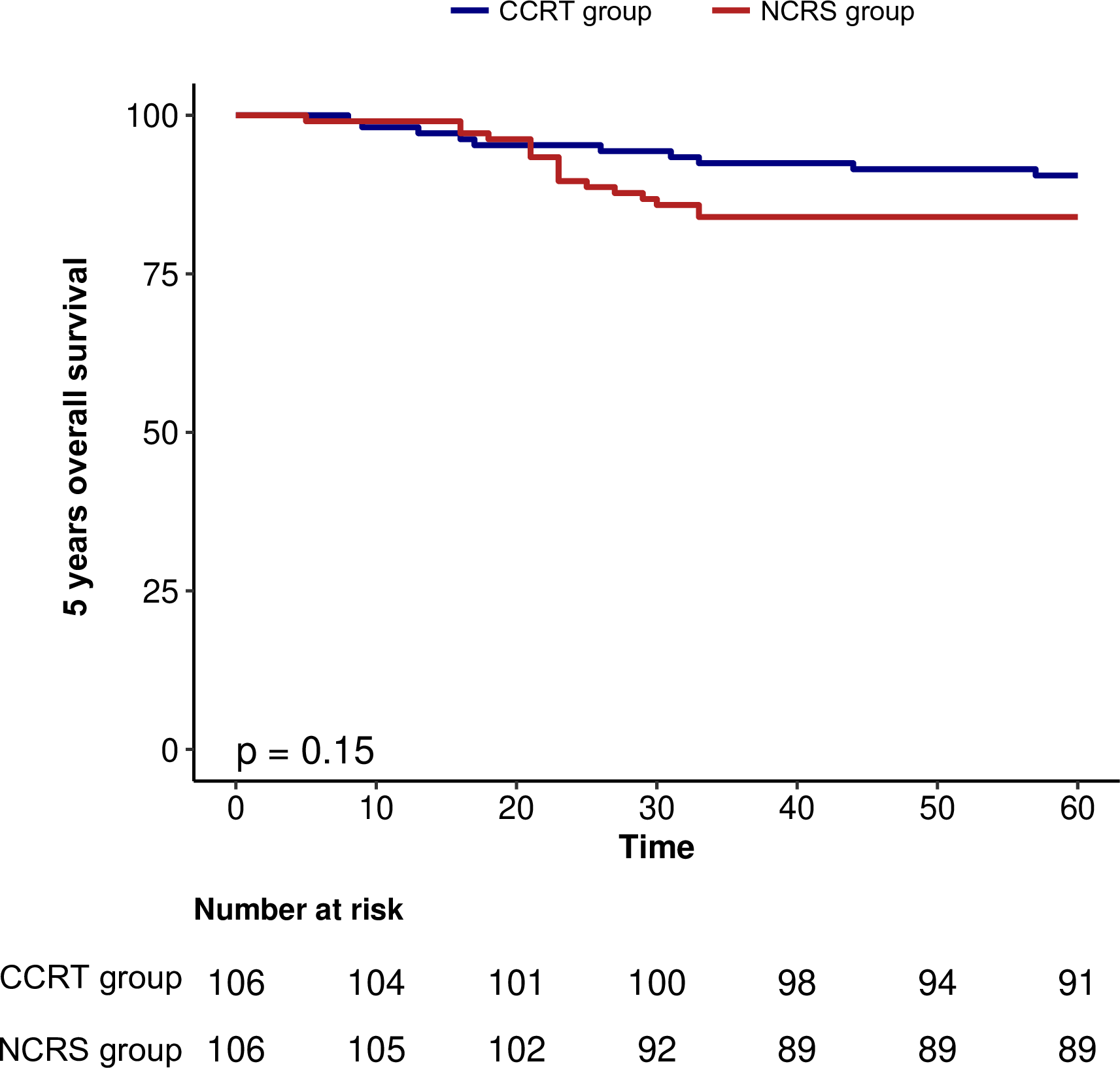

Supplement: S2 Fig — (TIF) [file pone.0319405.s002.tif]

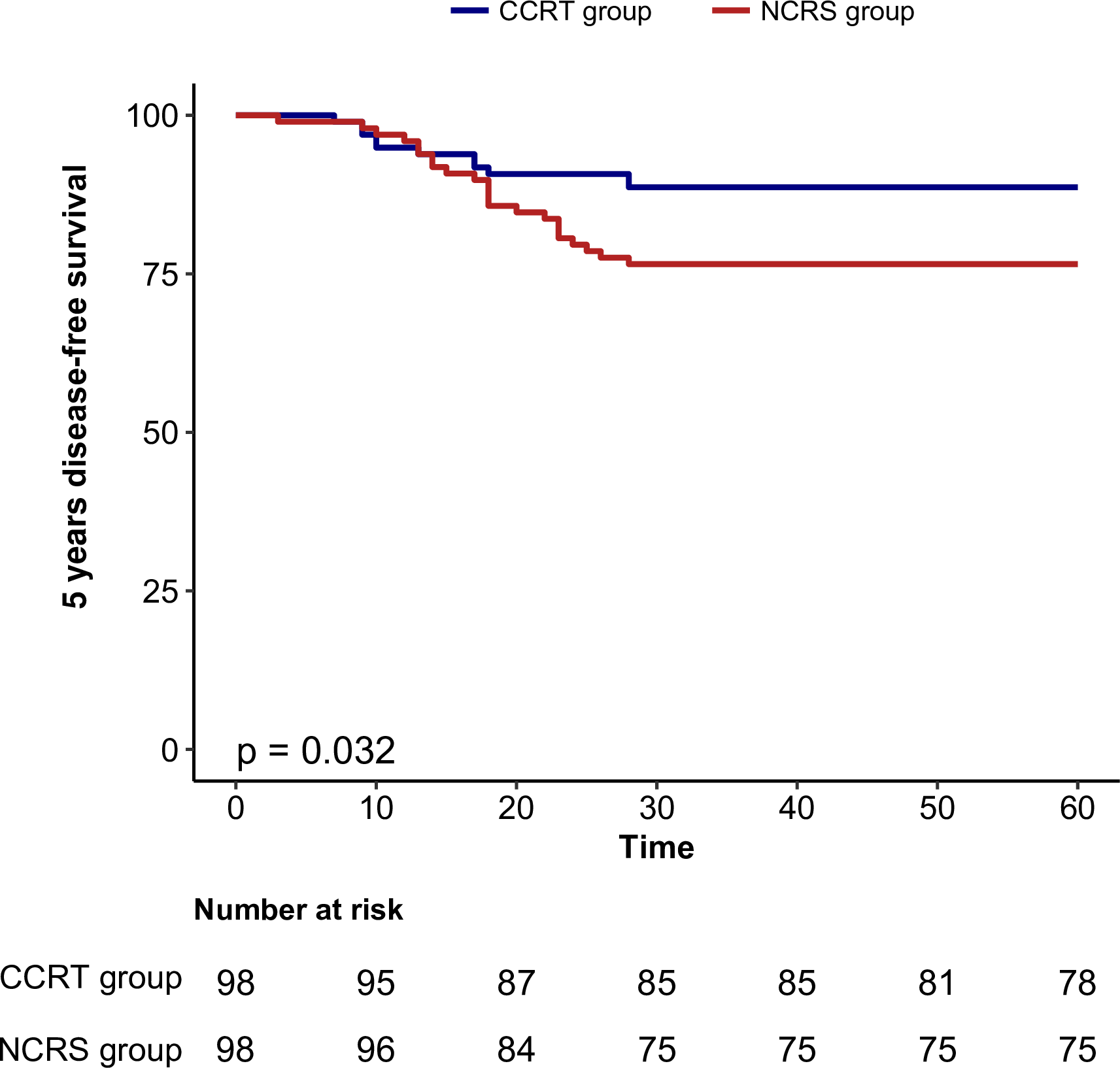

Supplement: S3 Fig — (TIF) [file pone.0319405.s003.tif]

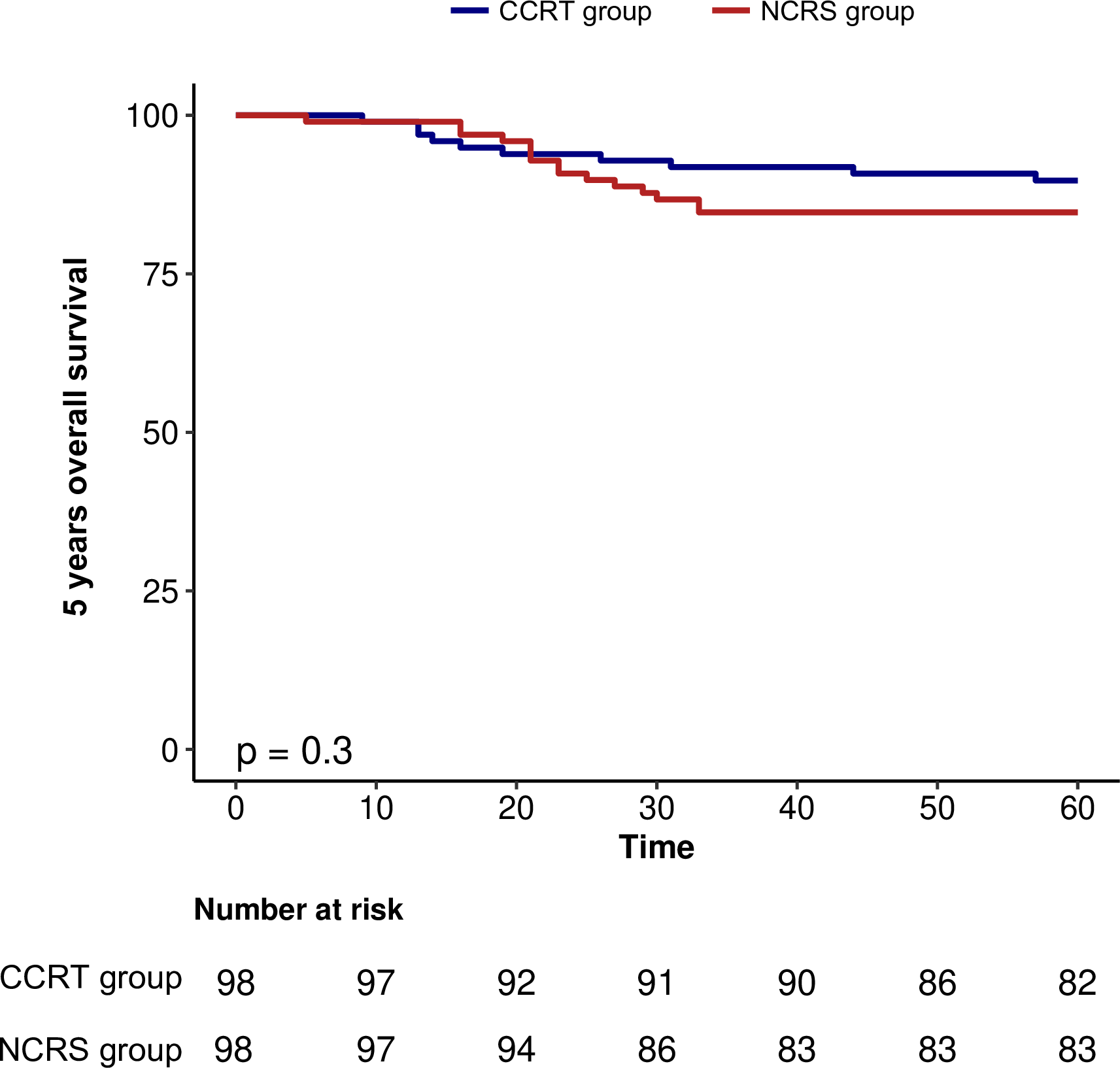

Supplement: S4 Fig — (TIF) [file pone.0319405.s004.tif]

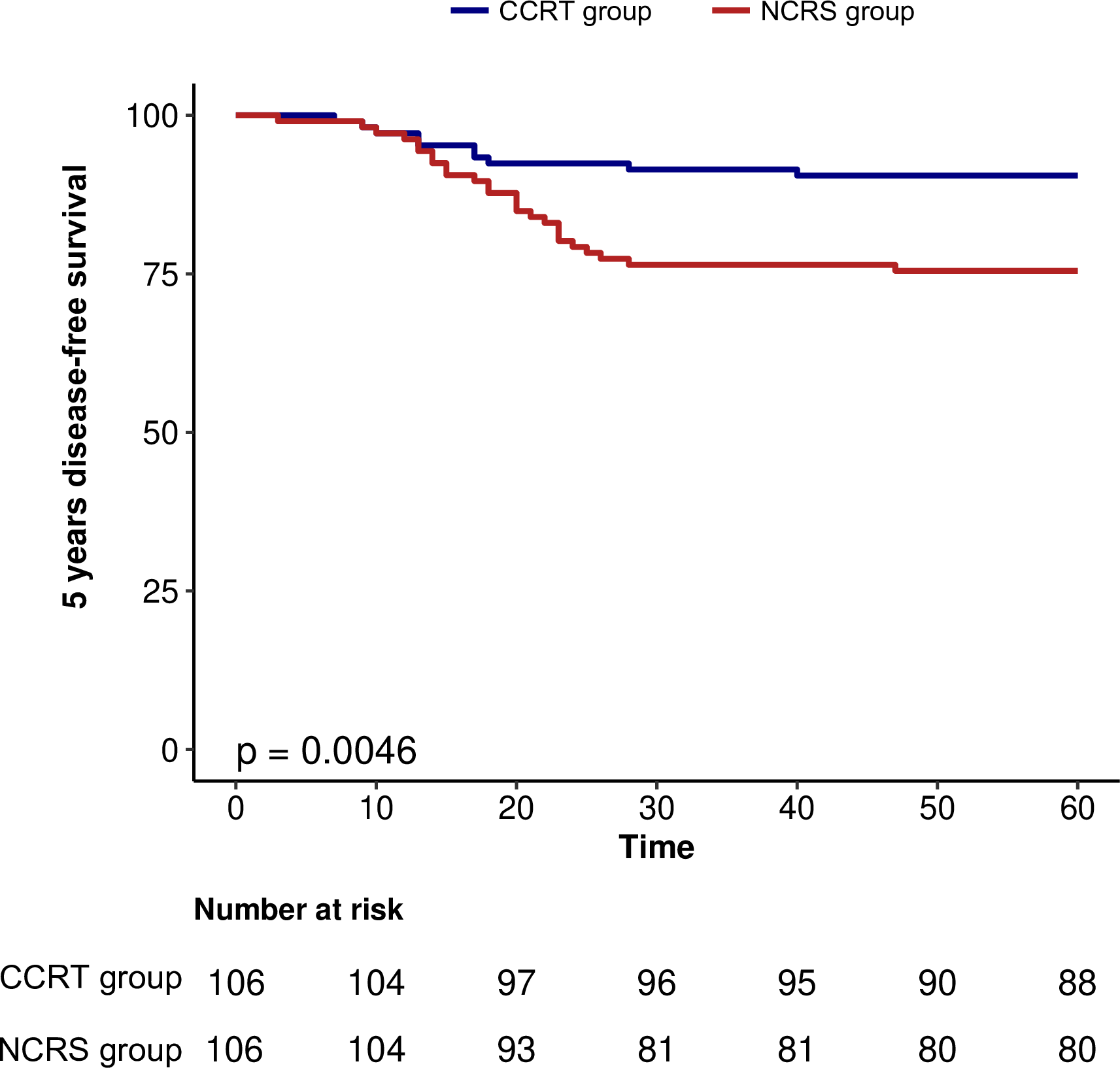

Supplement: S5 Fig — (TIF) [file pone.0319405.s005.tif]

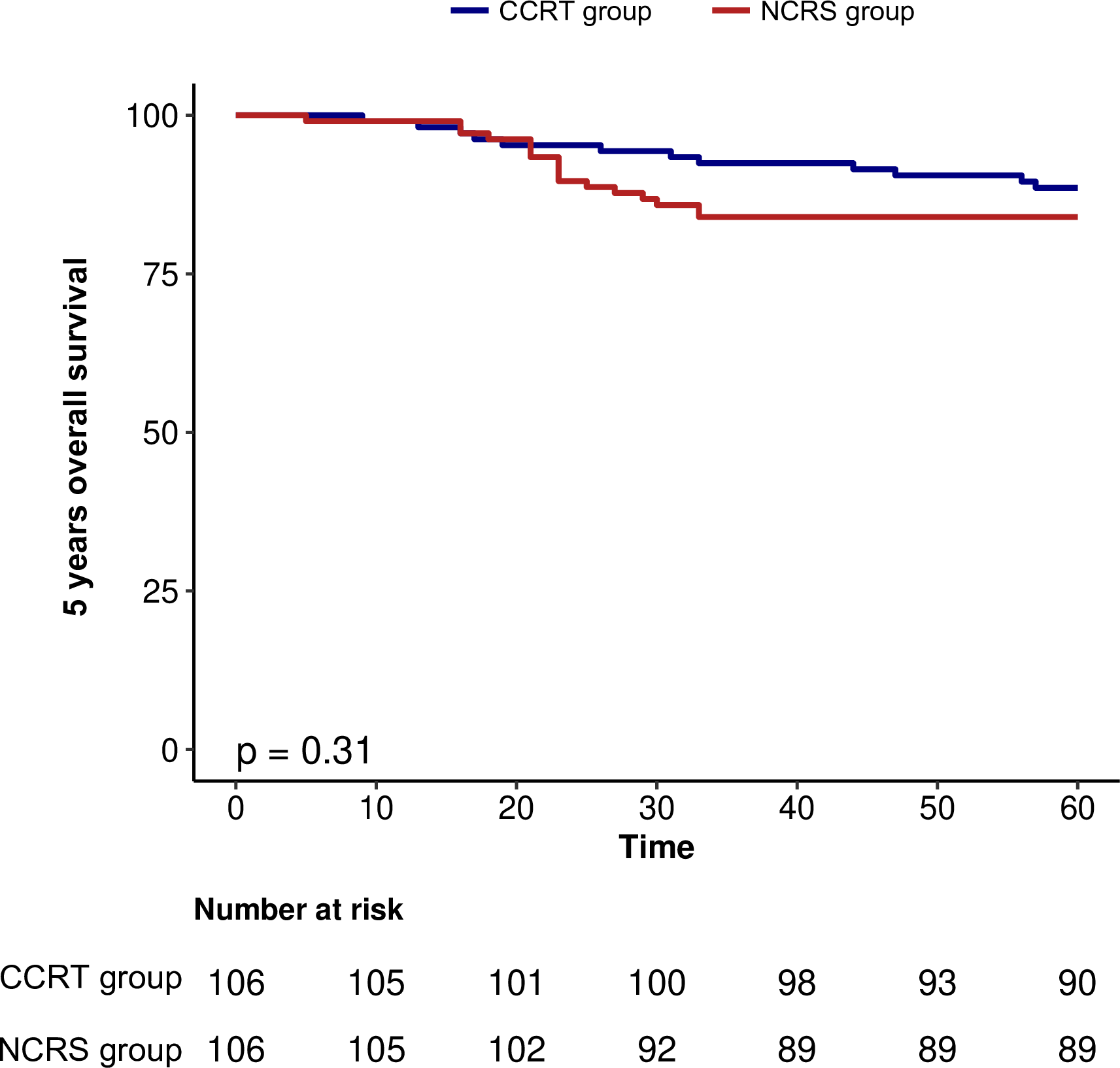

Supplement: S6 Fig — (TIF) [file pone.0319405.s006.tif]
