# Supplementary material for: A Comparison of neoadjuvant chemotherapy and concurrent chemoradiotherapy for for FIGO 2018 stage IB3/IIA2 Cervical squamous cell carcinoma: Long-term efficacy and safety in a resource-limited setting
Source: PLoS One. 2025 Mar 25;20(3):e0319405. doi: 10.1371/journal.pone.0319405 (PMC11936288; doi:10.1371/journal.pone.0319405)
Supplement: S1 Table — (DOCX) [file pone.0319405.s007.docx]

**Supplementary Table1.** Multivariate analyses of the OS rate and DFS rate by Cox proportional hazards regression models before and after PSM for patients with squamous or adenocarcinoma

| **Characteristic** | **Before matching** | | | | **After matching** | | | |
| --- | --- | --- | --- | --- | --- | --- | --- | --- |
|  | **OS** | | **DFS** | | **OS** | | **DFS** | |
|  | **aHR(95% CI)** | **p-value** | **aHR(95%CI)** | **p-value** | **aHR(95%CI)** | **p-value** | **aHR(95%CI)** | **p-value** |
| Age >46 years | 1.24 (0.67~2.29) | 0.5 | 0.67 (0.39~1.15) | 0.147 | 1.54 (0.7~3.42) | 0.287 | 0.71 (0.37~1.36) | 0.299 |
| Anemia before treatment | 0.89 (0.37~2.12) | 0.793 | 1.4 (0.71~2.78) | 0.335 | 1.27 (0.46~3.49) | 0.638 | 2.34 (1.09~5.03) | 0.029 |
| Initial tumor size >4.3 cm | 3.74 (1.41~9.9) | 0.008 | 2.47 (1.14~5.36) | 0.023 | 7.69 (1.74~34) | 0.007 | 3.17 (1.09~9.18) | 0.033 |
| Histologic grade G2-3 | 2.41 (1.13~5.14) | 0.023 | 2.29 (1.13~4.66) | 0.022 | 2.27 (0.55~9.3) | 0.256 | 2.13 (0.66~6.89) | 0.208 |
| FIGO 2018 stage (IB3 vs. IIA2) | 2.21 (1.02~4.82) | 0.045 | 5.59 (2.01~15.57) | 0.001 | 1.41 (0.53~3.78) | 0.496 | 5.49 (1.32~22.88) | 0.019 |
| Treatment (NCRS vs. CCRT) | 8.82 (3.01~25.86) | <0.001 | 3.3 (1.59~6.84) | 0.001 | 7.54 (2.04~27.9) | 0.002 | 2.97 (1.25~7.04) | 0.013 |

NCRS: Neoadjuvant chemotherapy followed by radical surgery; CCRT, concurrent chemoradiotherapy; FIGO: International Federation of Gynecology and Obstetrics;OS: overall survival; DFS: disease-free survival; aHR: adjust hazard radio; CI: confidence interval.
