# Supplementary material for: A Comparison of neoadjuvant chemotherapy and concurrent chemoradiotherapy for for FIGO 2018 stage IB3/IIA2 Cervical squamous cell carcinoma: Long-term efficacy and safety in a resource-limited setting
Source: PLoS One. 2025 Mar 25;20(3):e0319405. doi: 10.1371/journal.pone.0319405 (PMC11936288; doi:10.1371/journal.pone.0319405)
Supplement: S2 Table — (DOCX) [file pone.0319405.s008.docx]

**Supplementary Table 2.** Multivariate analyses of the OS rate and DFS rate by Cox proportional hazards regression models before and after PSM for patients with or without adjuvant therapy

| **Characteristic** | **Before matching** | | | | **After matching** | | | |
| --- | --- | --- | --- | --- | --- | --- | --- | --- |
|  | **OS** | | **DFS** | | **OS** | | **DFS** | |
|  | **aHR(95% CI)** | **p-value** | **aHR(95%CI)** | **p-value** | **aHR(95%CI)** | **p-value** | **aHR(95%CI)** | **p-value** |
| Age >46 years | 1.12 (0.59~2.13) | 0.738 | 2.06 (1.12~3.8) | 0.021 | 1.14 (0.51~2.52) | 0.748 | 0.64 (0.33~1.25) | 0.188 |
| Anemia before treatment | 0.64 (0.23~1.72) | 0.373 | 0.6 (0.33~1.08) | 0.09 | 0.52 (0.15~1.83) | 0.311 | 1.46 (0.65~3.27) | 0.361 |
| Initial tumor size >4.3 cm | 6.14 (2.06~18.27) | 0.001 | 1.4 (0.67~2.9) | 0.37 | 6.01 (1.22~29.75) | 0.028 | 2.3 (0.72~7.36) | 0.159 |
| Histologic grade G2-3 | 1.76 (0.77~4.06) | 0.183 | 2.45 (0.96~6.29) | 0.062 | 1.22 (0.45~3.32) | 0.7 | 4.56 (1.09~19.09) | 0.038 |
| FIGO 2018 stage (IB3 vs. IIA2) | 7.56 (2.48~23.06) | <0.001 | 5.55 (1.71~17.94) | 0.004 | 8.96 (1.91~42) | 0.005 | 2.77 (1.08~7.12) | 0.034 |
| Treatment (NCRS vs. CCRT) | 1.71 (0.87~3.39) | 0.122 | 3 (1.31~6.9) | 0.01 | 1.74 (0.78~3.89) | 0.174 | 2.23 (1.11~4.48) | 0.024 |

NCRS: Neoadjuvant chemotherapy followed by radical surgery; CCRT, concurrent chemoradiotherapy; FIGO: International Federation of Gynecology and Obstetrics;OS: overall survival; DFS: disease-free survival; aHR: adjust hazard radio; CI: confidence interval.
