# Supplementary material for: A Comparison of neoadjuvant chemotherapy and concurrent chemoradiotherapy for for FIGO 2018 stage IB3/IIA2 Cervical squamous cell carcinoma: Long-term efficacy and safety in a resource-limited setting
Source: PLoS One. 2025 Mar 25;20(3):e0319405. doi: 10.1371/journal.pone.0319405 (PMC11936288; doi:10.1371/journal.pone.0319405)
Supplement: S3 Table — (DOCX) [file pone.0319405.s009.docx]

**Supplementary Table 3.** Multivariate analyses of the OS rate and DFS rate by Cox proportional hazards regression models before and after PSM for patients with all pathology types

| **Characteristic** | **Before matching** | | | | **After matching** | | | |
| --- | --- | --- | --- | --- | --- | --- | --- | --- |
|  | **OS** | | **DFS** | | **OS** | | **DFS** | |
|  | **aHR(95% CI)** | **p-value** | **aHR(95%CI)** | **p-value** | **aHR(95%CI)** | **p-value** | **aHR(95%CI)** | **p-value** |
| Age >46 years | 1.13 (0.62~2.06) | 0.68 | 0.64 (0.38~1.1) | 0.105 | 1.72 (0.8~3.72) | 0.168 | 0.88 (0.46~1.67) | 0.686 |
| Anemia before treatment | 0.87 (0.36~2.07) | 0.75 | 1.38 (0.7~2.75) | 0.353 | 0.79 (0.26~2.34) | 0.668 | 1.75 (0.8~3.83) | 0.158 |
| Initial tumor size >4.3 cm | 3.61 (1.38~9.43) | 0.009 | 2.44 (1.13~5.24) | 0.022 | 10.39 (2.29~47.17) | 0.002 | 4.15 (1.44~12.02) | 0.009 |
| Histologic grade G2-3 | 2.6 (1.28~5.29) | 0.008 | 2.4 (1.23~4.69) | 0.01 | 1.64 (0.41~6.56) | 0.487 | 1.7 (0.53~5.47) | 0.374 |
| FIGO 2018 stage (IB3 vs. IIA2) | 2.25 (1.04~4.88) | 0.04 | 5.69 (2.05~15.8) | 0.001 | 1.45 (0.54~3.89) | 0.456 | 5.02 (1.2~20.97) | 0.027 |
| Treatment (NCRS vs. CCRT) | 8.98 (3.1~26) | <0.001 | 3.38 (1.65~6.94) | 0.001 | 8.6 (2.25~32.97) | 0.002 | 2.79 (1.17~6.64) | 0.021 |

NCRS: Neoadjuvant chemotherapy followed by radical surgery; CCRT, concurrent chemoradiotherapy; FIGO: International Federation of Gynecology and Obstetrics;OS: overall survival; DFS: disease-free survival; aHR: adjust hazard radio; CI: confidence interval.
